# Esculentin-2CHa-Related Peptides Modulate Islet Cell Function and Improve Glucose Tolerance in Mice with Diet-Induced Obesity and Insulin Resistance

**DOI:** 10.1371/journal.pone.0141549

**Published:** 2015-10-29

**Authors:** Opeolu O. Ojo, Dinesh K. Srinivasan, Bosede O. Owolabi, Srividya Vasu, J. Michael Conlon, Peter R. Flatt, Yasser H. A. Abdel-Wahab

**Affiliations:** 1 SAAD Centre for Pharmacy & Diabetes, School of Biomedical Sciences, University of Ulster, Coleraine, BT52 1SA, United Kingdom; 2 School of Sport, Health and Bioscience, University of East London, Stratford, E15 4LZ, United Kingdom; Tohoku University, JAPAN

## Abstract

The frog skin host-defense peptide esculentin-2CHa (GFSSIFRGVA^10^KFASKGLGK D^20^LAKLGVDLVA^30^CKISKQC) displays antimicrobial, antitumor, and immunomodulatory properties. This study investigated the antidiabetic actions of the peptide and selected analogues. Esculentin-2CHa stimulated insulin secretion from rat BRIN-BD11 clonal pancreatic β-cells at concentrations greater than 0.3 nM without cytotoxicity by a mechanism involving membrane depolarization and increase of intracellular Ca^2+^. Insulinotropic activity was attenuated by activation of K_ATP_ channels, inhibition of voltage-dependent Ca^2+^ channels and chelation of extracellular Ca^2+^. The [L21K], [L24K], [D20K, D27K] and [C31S,C37S] analogues were more potent but less effective than esculentin-2CHa whereas the [L28K] and [C31K] analogues were both more potent and produced a significantly (P < 0.001) greater maximum response. Acute administration of [L28K]esculentin-2CHa (75 nmol/kg body weight) to high fat fed mice with obesity and insulin resistance enhanced glucose tolerance and insulin secretion. Twice-daily administration of this dose of [L28K]esculentin-2CHa for 28 days had no significant effect on body weight, food intake, indirect calorimetry or body composition. However, mice exhibited decreased non-fasting plasma glucose (P < 0.05), increased non-fasting plasma insulin (P < 0.05) as well as improved glucose tolerance and insulin secretion (P < 0.01) following both oral and intraperitoneal glucose loads. Impaired responses of isolated islets from high fat fed mice to established insulin secretagogues were restored by [L28K]esculentin-2CHa treatment. Peptide treatment was accompanied by significantly lower plasma and pancreatic glucagon levels and normalization of α-cell mass. Circulating triglyceride concentrations were decreased but plasma cholesterol and LDL concentrations were not significantly affected. The data encourage further investigation of the potential of esculentin-2CHa related peptides for treatment of patients with type 2 diabetes.

## Introduction

Skin secretions of many species of Anura (frogs and toads) represent a valuable source of peptides with therapeutic potential. These peptides, whose primary function is believed to be host-defence, are best known for their antimicrobial properties and there are many examples of components with potent activity against multidrug-resistant pathogenic Gram-positive and Gram-negative bacteria and fungi (reviewed in [1–4]). However, frog skin host-defense peptides are multifunctional and may also display anti-tumor, antiviral, immunomodulatory, and chemoattractive activities (reviewed in [5]). In addition, myotropic peptides produced in the skin, such as those related to mammalian tachykinins, bradykinin and CCK-8, may play a role in deterring ingestion by predators [6]. There are no conserved structural domains in amphibian host-defense peptides that are responsible for biological activity but with few exceptions they are cationic and have the propensity to adopt an amphipathic, α-helical conformation in a membrane-mimetic solvent or in the environment of a phospholipid vesicle [2].

One such host-defense peptide with therapeutic potential is esculentin-2CHa (GFSSIFRGVAKFASKGLGKDLAKLGVDLVACKISKQC), first isolated from norepinephrine-stimulated skin secretions of the Chiricahua leopard frog *Lithobates chiricahuensis* (Ranidae) [7]. This compound shows potent broad-spectrum antimicrobial properties including activity against clinical isolates of multidrug-resistant strains of *Staphylococcus aureus*, *Acinetobacter baumannii*, and *Stenotrophomonas maltophilia*. The peptide also stimulates the release of the anti-inflammatory cytokine IL-10 by mouse lymphoid cells and displays high cytotoxic potency against human non-small cell lung adenocarcinoma A549 cells but relatively low hemolytic activity against human erythrocytes [8]. Interestingly, such actions on cytokine production by mouse lymphoid cells were also extended to TNF-alpha which may impact on beta cells [8]. Structure-activity studies indicate that removal of the either hydrophobic N-terminal hexapeptide (GFSSIF) or the cyclic C-terminal domain (CKISKQC) and replacement of the Cys^31^ and Cys^37^ residues by serine results in appreciable decreases in cytotoxicity against microorganisms and mammalian cells. In contrast, increasing cationicity by substitution of the Asp^20^ and Asp^27^ residues by L-Lysine resulted in a modest increase in potency against all microorganisms tested (up to 4-fold) [8].

The current pandemic of type 2 diabetes has necessitated a search for new types of therapeutic agents and naturally occurring incretin peptides that stimulate insulin release in response to high circulating glucose concentration are receiving increasing attention. Several long-acting analogues of the potent incretin GLP-1 are currently in clinical use [9]. A number of frog skin peptides that were first identified on the basis of their ability to inhibit the growth of microorganisms have subsequently been shown to possess the ability to release insulin from the BRIN-BD11 clonal β-cells and isolated mouse islets at low concentrations that are not cytotoxic to the cells and to improve glucose tolerance in mice following acute administration (reviewed in [5, 10]). The high fat fed mouse exhibits obesity, glucose intolerance and insulin resistance and so is a useful model for a preliminary investigation of the therapeutic potential of peptides in treatment of patients with type 2 diabetes [11]. More recently, it has been shown that twice daily treatment of high-fat fed mice for up to 28 days with tigerinin-1R [12], magainin-AM1 [13], and CPF-SE1 [14] results in an improvement in glucose tolerance, insulin sensitivity, and islet β-cell secretory responsiveness.

In the present study, the antidiabetic potential of esculentin-2CHa and selected analogues with increased cationicity (Table 1) was assessed *in vitro* using BRIN-BD11 cells and isolated mouse islets and *in vivo* in studies using the high fat fed mouse.

**Table 1:** Amino acid sequence and physicochemical properties of esculentin-2CHa and its substituted analogues.

Peptide	Sequence	Net charge at pH 7	Isoelectric point
Esculentin-2CHa	GFSSIFRGVAKFASKGLGKDLAKLGVDLVACKISKQC	5	10.82
[L21K]Esculentin-2CHa	GFSSIFRGVAKFASKGLGKD**K**AKLGVDLVACKISKQC	6	10.91
[L24K]Esculentin-2CHa	GFSSIFRGVAKFASKGLGKDLAK**K**GVDLVACKISKQC	6	10.91
[L28K]Esculentin-2CHa	GFSSIFRGVAKFASKGLGKDLAKLGVD**K**VACKISKQC	6	10.91
[C31K]Esculentin-2CHa	GFSSIFRGVAKFASKGLGKDLAKLGVDLVA**K**KISKQC	6	10.65
[C31S,C37S]Esculentin-2CHa	GFSSIFRGVAKFASKGLGKDLAKLGVDLVA**S**KISKQ**S**	5	10.82
[D20K, Lys27]Esculentin-2CHa	GFSSIFRGVAKFASKGLGK**K**LAKLGV**K**LVACKISKQC	9	11.97

## Materials and Methods

### Peptide synthesis and purification

Synthetic esculentin-2CHa and its analogues (Table 1) were purchased in crude form GL Biochem Ltd (Shanghai, China) and purified to near homogeneity (> 98% pure) by reversed-phase HLPC on a (2.2 cm x 25 cm) Vydac 218TP1022 (C18) column equilibrated with acetonitrile/water/triflouroacetic acid (21.0/78.9/0.1 v/v) mobile phase at a flow rate of 6 ml/min. The concentration of acetonitrile in the eluting buffer was raised to 56% (v/v) over 60 min. The molecular masses of the peptides were confirmed using MALDI-TOF mass spectrometry.

### *In vitro* insulin-releasing studies

*In vitro* insulin-releasing effects of esculentin-2CHa and its analogues were assessed using BRIN-BD11 rat clonal β-cells and mouse islets. In the first set of experiments, BRIN-BD11 cells were incubated with the peptides in the concentration range (1 pM– 3 μM) in Krebs-Ringer bicarbonate buffer containing 5.6 mM glucose for 20 min at 37°C as previously described [15–17]. Insulin-releasing effects were also assessed using buffer supplemented with 1.4 and 16.7mm glucose concentrations and with established modulators of insulin release as previously described [15–17]. In a second set of experiments, islets from NIH Swiss mice, isolated by collagenase digestion [18,19], were incubated with a range of concentrations of esculentin-2CHa or its analogues (0.1 nM– 1 μM) for 1 h in Krebs-Ringer bicarbonate (KRB) buffer supplemented with 1.4, 5.6 or 16.7 mM glucose. Following test incubations, aliquots of buffer were retrieved and stored at -20°C for measurement of insulin by radioimmunoassay [20]. In the experiments with BRIN-BD11 cells, the release of lactate dehydrogenase (LDH) was measured as an indicator of the integrity of the plasma membrane using commercially available CytoTox 96 non-radioactive cytotoxicity assay kit (Promega, Madison, WI, USA) according to the manufacturer’s recommended protocol.

### Membrane potential studies and intracellular calcium ([Ca^2+^]_i_)

Effects of esculentin-2CHa (1 μM) and its analogues on membrane potential and [Ca^2+^]_i_ in BRIN-BD11 cells over a period of 5 min were assessed using commercially available FLIPR membrane or calcium assay kit (Molecular Devices, USA) as previously described [15]. Data were captured using a Flexstation 3 microplate reader equipped with automatic fluid transfer unit (Molecular Devices, USA).

### Laboratory animals

Male National Institutes of Health (NIH) Swiss mice (Harlan Ltd, UK) were housed separately in an air-conditioned room (22 ± 2°C) with relative humidity of 51 ± 5% and a 12-hour light: 12-hour dark cycle and maintained on a high fat diet containing 45% fat, 20% protein and 35% carbohydrate (total energy 19.5 kJ/g, Dietex International Ltd, Witham, UK) for 120 days. Mice maintained on standard rodent diet containing 10% fat, 30% protein and 60% carbohydrates (total energy 12.99 kJ/g) were used as controls. Both groups of animals were allowed food and water *ad libitum*. All animal experiments were carried out in accordance with the UK Animals (Scientific Procedures) Act 1986 and EU Directive 2010/63EU for animal experiments. The study was approved by University of Ulster Research Ethics Committee.

### Acute *in vivo* studies

For acute *in vivo* studies, overnight fasted, high fat fed mice (n = 8) received an intraperitoneal injection of glucose alone (18 mmol/kg body weight) or in combination with esculentin-2CHa or its analogues (75 nmol/kg body weight). This peptide dose was selected on the basis of a pilot study that examined acute effects of different doses of esculentin-2CHa on glucose tolerance in lean mice. Blood samples were collected as described previously before injection and at times indicated in the Figures for the measurement of plasma glucose and insulin concentrations.

### Longer-term *in vivo* studies

High-fat fed mice with clearly manifested features of obesity and hyperglycaemia received twice daily injections of either saline vehicle (0.9% (w/v) (high fat fed controls) or [L28K]esculentin-2CHa (75 nmol/kg body weight) for 28 days. This peptide was chosen based on its potent *in vitro* and acute *in vivo* actions. Mice (n = 8) fed standard rodent diet and injected with saline were used as lean controls. Energy intake, bodyweight, non-fasting blood glucose and plasma insulin concentrations were monitored every 72 h throughout the duration of the study. At the end of the 28 day treatment period, glucose tolerance (18 mmol/kg body weight, intraperitoneal or oral, overnight fasted) and insulin sensitivity (25 U/kg body weight) were assessed as previously described [12–14]. Indirect calorimetry and energy expenditure in treated and control mice were measured using the Comprehensive Laboratory Animal Monitoring System (CLAMS) metabolic chambers (Columbus Instruments, Columbus, OH, USA). Total body lean and fat mass, bone mineral density and bone mineral content were also measured using DXA scanning (Piximus Densitometer, USA) [21]. Improvement in beta cell function in [L28K]esculentin-2CHa-treated and control mice were evaluated from the insulin secretory responses of islets isolated from these animals to established insulin secretagogues and incretin hormones (16.7 mM glucose, 1 μM GLP-1, 1 μM GIP, 10 mM alanine, 10 mM arginine and 30 mM KCl). Changes in islet morphology were assessed using pancreatic tissues excised from mice treated with [L28K]esculentin-2CHa or saline for 28 days as previously described [12–14].

### Biochemical measurements

Pancreatic tissues were homogenized in 20 mM Tris-HCl, 150 mM NaCl, 1 mM EDTA, 1 mM EGTA and 0.5% Triton X 100; pH 7.5 as previously described [14]. Blood samples (approximately 150 μl), collected from the cut tip of the tail vein of unanesthetized mice at intervals indicated in the Figures were used for blood glucose measurements and determination of plasma insulin as previously described [13,14]. Blood glucose was measured using a hand-held Ascencia Contour meter (Bayer Healthcare, UK). Plasma and pancreatic insulin were determined by radioimmunoassay [20]. Plasma and pancreatic glucagon contents were determined by ELISA using commercially available kit (Millipore, MA, USA). Plasma creatinine, alanine transaminase (ALT), aspartic acid transaminase (AST) and alkaline phosphatase (ALP) were measured using commercially available kits (Randox Laboratories, UK) as indicators of renal and liver function. Plasma triglyceride and cholesterol concentrations were measured using an automated clinical chemistry analyser (I-lab 650, and reagents purchased from Instrumentation Laboratory (Warrington, UK). The assay kit for cholesterol was obtained from Randox Laboratories (Antrim, UK). Estimation of LDL cholesterol concentrations was achieved using the Friedewald equation as described previously [22].

### Statistical analysis

Results are expressed as mean ± S.E.M. Values were compared using one-way ANOVA followed by Student-Newman-Keuls *post hoc* test. Area under the curve (AUC) analysis was performed using the trapezoidal rule with baseline correction. P < 0.05 was considered statistically significant.

## Results

### *In vitro* insulin releasing effects

**Fig 1:**
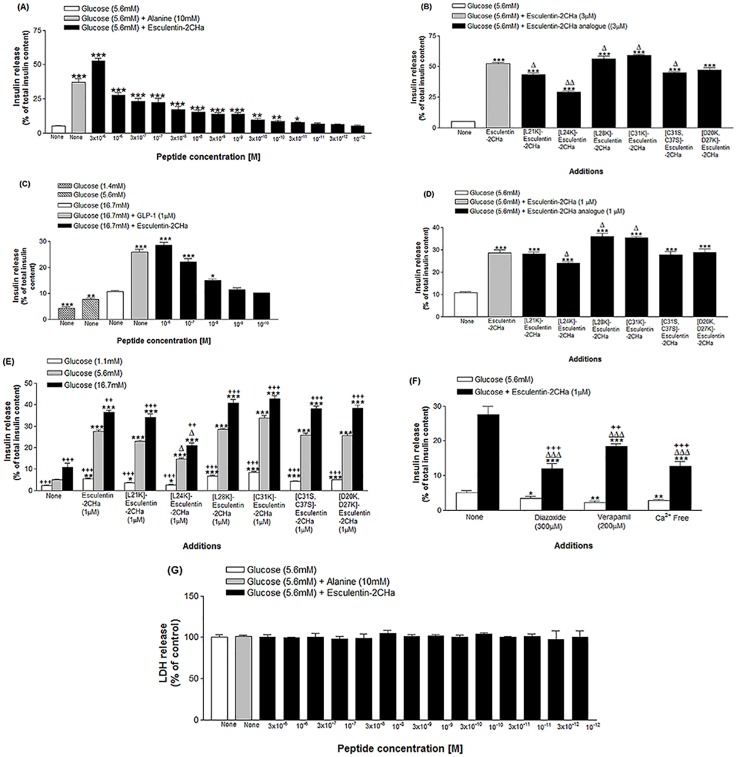
Dose dependent effects of esculentin-2CHa and its substituted analogues on insulin release from BRIN-BD11 cells (A, B, E, F) and mouse islets (C, D). Effects on lactate dehydrogenase (LDH) release (G) is also shown. Values are mean ± SEM with n = 8. For A, B, D and F, *P < 0.05, **P < 0.01, ***P < 0.001 compared with 5.6mM glucose. For B and D, ^Δ^P < 0.05, ^ΔΔ^P < 0.01 compared with native esculentin-2CHa. For C, *P < 0.05, **P < 0.01, ***P < 0.001 compared with 16.7 mM glucose. For E, *P < 0.05, **P < 0.01, ***P < 0.001 compared with the same concentration of glucose alone. ^Δ^P<0.05 compared with esculentin-2CHa at the same glucose concentration. ^++^P < 0.01, ^+++^P < 0.001 compared with incubation at 5.6 mM glucose for each peptide. For F, ^ΔΔΔ^P < 0.001 compared with respective incubation in the absence of the peptide. ^+^P < 0.05, ^++^P < 0.01, ^+++^P < 0.001 compared with incubation at 5.6 mM glucose in the presence of esculentin-2CHa alone.

The basal rate of insulin secretion from BRIN BD11 cells at 5.6 mM glucose was about 5% of total insulin content of one million cells in 20 min. This was increased by 7.3-fold in the presence of alanine (10mM) (P<0.001; n = 8) (Fig 1A). Esculentin-2CHa produced a significant (P < 0.05) and concentration-dependent stimulation of insulin release at concentrations ≥ 0.03nM. At the highest concentration of 3μM, the peptide produced a near maximum 10.3-fold insulin-secretory response (P < 0.001). All analogues of esculentin-2CHa tested produced a significant (P < 0.05) and concentration-dependent stimulation of insulin release from BRIN-BD11 cells (Fig 1B). The threshold concentration (minimum concentration producing a significant increase in the rate of insulin release) of all analogues, except [D20K,D27K]esculentin-2CHa, was 0.01 nM. Stimulatory effects at 3 μM of [L28K]- and [C31S]-esculentin-2CHa were significantly (P < 0.05) greater than those of the native peptide and the other analogues (Table 2).

**Table 2:** Effects of esculentin-2CHa and its analogues on insulin- and LDH-release, membrane potential and intracellular calcium concentration in BRIN-BD11 cells.

Peptide	BRIN-BD11 cells (Threshold concentration)	Mouse islets (Threshold concentration)	LDH release (% of control)	Membrane potential (AUC, x 10^3^ RFU)	Intracellular Ca^2+^ (AUC, x 10^2^ RFU)
Control (Glucose 5.6mM)	**-**	**-**	100.0±2.9	3.71±1.05	4.30±0.40
Esculentin-2CHa	0.3nM	10nM	102.7±1.1	8.01±0.28**	6.44±0.47*
[L21K]Esculentin-2CHa	0.01nM	10nM	100.6±2.0	7.78±0.31**	6.14±0.41*
[L24K]Esculentin-2CHa	0.01nM	10nM	101.3±3.1	7.87±0.19**	6.28±0.62*
[L28K]Esculentin-2CHa	0.01nM	1nM	101.8±1.5	7.78±0.22**	7.28±0.94*
[C31K]Esculentin-2CHa	0.01nM	0.1nM	100.2±2.8	7.92±0.14**	6.32±0.72*
[C31S, C37S]Esculentin-2CHa	0.01nM	1nM	104.3±3.1	7.96±0.24**	6.55±0.36*
[D20K, D27K]Esculentin-2CHa	0.1nM	10nM	102.5±2.6	7.85±0.32**	6.10±0.46*

LDH release data were obtained at the highest peptide concentration (3 μM). Membrane potential and intracellular Ca^2+^ experiments were carried out at 1μM concentration. Values are mean ± SEM with n = 8.

*P < 0.05

**P < 0.01 compared with 5.6mM glucose.

The insulinotropic actions of esculentin-2CHa and its analogues were replicated in incubations performed with isolated mouse islets (Fig 1C and 1D). Basal insulin release from islets at 16.7 mM glucose was increased 2.4-fold by GLP-1 (10^−6^ M). A similar threshold concentration was observed for esculentin-2CHa and its [L21K], [L24K] and [D20K, D27K] analogues at 16.7 mM glucose (Table 2). However, other analogues stimulated insulin secretion from islets at concentrations ≥ 1 nM. The stimulation produced by the highest concentration of esculentin-2CHa (1 μM, 2.7-fold, P < 0.001) was comparable to the insulinotropic effect exhibited by the same concentration (1 μM) of its [L21K] (2.6-fold, P < 0.001), [C31S, C37S] (2.6-fold, P < 0.001) and [D20K,D27K] analogues (1.7-fold, P < 0.001) but was significantly (P < 0.05) higher that the effect observed for [L24K]-esculentin-2CHa (1 μM, 2.3-fold) under the same experimental conditions. The stimulatory effects of the [L28K] (1 μM, 3.4-fold, P < 0.001) and [L31K] (1 μM, 3.3-fold, P < 0.001) analogues on isolated islets were significantly higher when compared with the native peptide (Table 2).

The stimulatory effects of esculentin-2CHa (1 μM) and its substituted analogues on BRIN-BD11 cells increased progressively as the concentration of glucose in the incubation buffer was increased from 1.1 to 16.7mM (Fig 1E). However, the stimulatory action of esculentin-2CHa (Fig 1F) and its analogues were significantly inhibited by diazoxide (300 μM, 52–57%, P < 0.001), verapamil (50 μM, 31–35%, P < 0.001) and by the absence of extracellular calcium (49–55%, P<0.001). No significant release of lactate dehydrogenase (LDH) from BRIN-BD11 cells was observed following incubation with esculentin-2CHa or any analogue tested (Fig 1G).

### Effects on membrane potential and intracellular Ca^2+^ concentration

**Fig 2:**
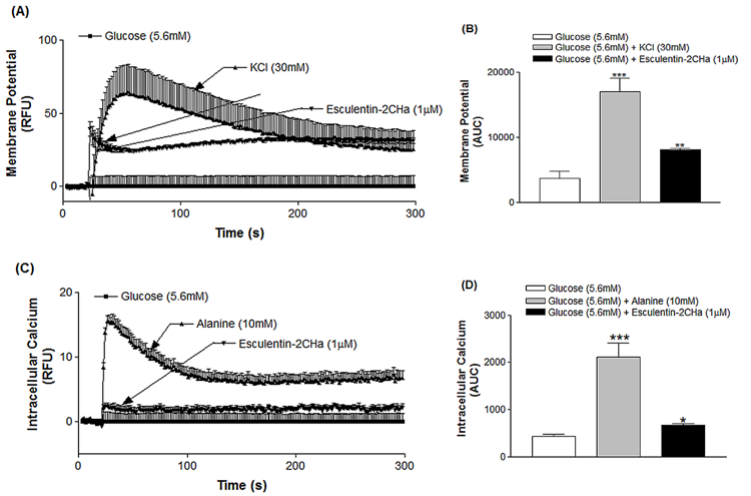
Effects of esculentin-2CHa on membrane potential (A, B) and intracellular calcium concentration (C, D) in BRIN-BD11 cells expressed as relative fluorescence unit, RFU (A, C) and area under the curve, AUC (B, D). Cells were incubated with esculentin-2CHa (1 μM) or its substituted analogue and data were collected every 1.52 s over a period of 5 min. Values are mean ± SEM with n = 6. *P < 0.05, **P < 0.01, ***P < 0.001 compared with 5.6 mM glucose.

Membrane potential of BRIN BD11 cells was increased by 5.2-fold (P < 0.001) in the presence of KCl (30 mM, Fig 2A and 2B). Esculentin-2CHa (1 μM) and its analogues also depolarized BRIN-BD11 cells, producing a 2.1-fold (P < 0.001) increase of membrane potential compared with 5.6mM glucose (Table 2). While alanine (10 mM) produced a marked increase (4.6-fold, P < 0.001) in the concentration of intracellular Ca^2+^, esculentin-2CHa and its analogues elicited only a modest response (1 μM, 1.5-fold, P < 0.05, Fig 2C and 2D, Table 2).

### Acute *in vivo* effects on glucose tolerance and insulin release

**Fig 3:**
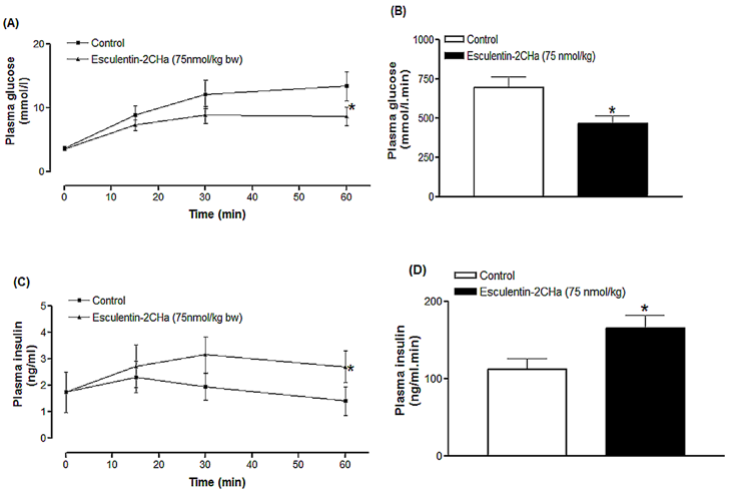
Effects of esculentin-2CHa on glucose tolerance (A, B) and plasma insulin response to glucose (C, D) in high fat fed mice expressed as line graph (A, C) and area under the curve, AUC (B, D). Values are mean ± SEM with n = 8. *P < 0.05 compared with control

Acute *in vivo* effects of esculentin-2CHa and its analogues were assessed in high fat mice with obesity-diabetes. Intraperitoneal administration of esculentin-2CHa (75 nmol/kg body weight) with glucose (18 mmol/kg body weight) significantly decreased the plasma glucose excursion compared with injection of glucose alone (Fig 3A). Integrated responses, presented as area under the glucose curve, showed that plasma glucose was 33% (P < 0.05) lower in mice injected with esculentin-2CHa (Fig 3B). This was accompanied by a 48% (P < 0.01, Fig 3C and 3D) increase in the integrated insulin response. The plasma glucose response was reduced by 4–52% (P ≤ 0.05) using different analogues of esculentin-2CHa, with the [L28K] and [C31K] peptides producing 52% (P < 0.01) and 40% (P < 0.05) decreases respectively (Table 3). A similar trend was observed for acute effects of the analogues on insulin secretion in high fat fed mice. Plasma insulin response was increased by 10–134% in mice treated with the [L28K] and [C31K] analogues producing stimulating responses that were 2.8- and 2.4-fold greater than that observed with esculentin-2CHa (Table 3).

**Table 3:** Acute effects of esculentin-2CHa and its analogues on glucose tolerance and insulin release in high fat fed mice.

Peptide	Plasma glucose (AUC, mmol/l.min)	Plasma insulin (AUC, ng/ml.min)
Control	693.53±71.34	111.86±14.03
Esculentin-2CHa	463.99±46.95*	165.05±16.89*
[L21K]Esculentin-2CHa	598.68±35.23^ΔΔ^	131.12±14.25^Δ^
[L24K]Esculentin-2CHa	662.25±32.14^ΔΔ^	129.89±11.02^Δ^
[L28K]Esculentin-2CHa	327.61±36.87** ^, Δ^	262.81±30.56** ^,ΔΔΔ^
[L31K]Esculentin-2CHa	410.72±29.25*	240.32±31.04** ^,ΔΔΔ^
[C31S, C37S]Esculentin-2CHa	618.65±86.0^ΔΔ^	122.74±28.65^Δ^
[D20K, D27K]Esculentin-2CHa	667.78±60.23^ΔΔ^	122.96±16.76^Δ^

Plasma glucose and insulin were measured prior to and after intraperitoneal administration of high fat fed mice with glucose (18 mmol/kg) alone (control) or in combination with peptide (75 nmol/kg body weight). Integrated responses to are presented as area under the curve values (AUC) for data collected at 0, 15, 30 and 60 min after peptide administration. Values are mean ± SEM with n = 8.

*P < 0.05

**P < 0.01 compared with control 5.6mM glucose. ^Δ^P < 0.05, ^ΔΔ^P<0.01, ^ΔΔΔ^P<0.001 compared with mice injected with esculentin-2CHa.

### Effects of 28 day administration of [L28K]esculentin-2CHa on body weight, food intake and non-fasting plasma glucose and insulin concentrations

**Fig 4:**
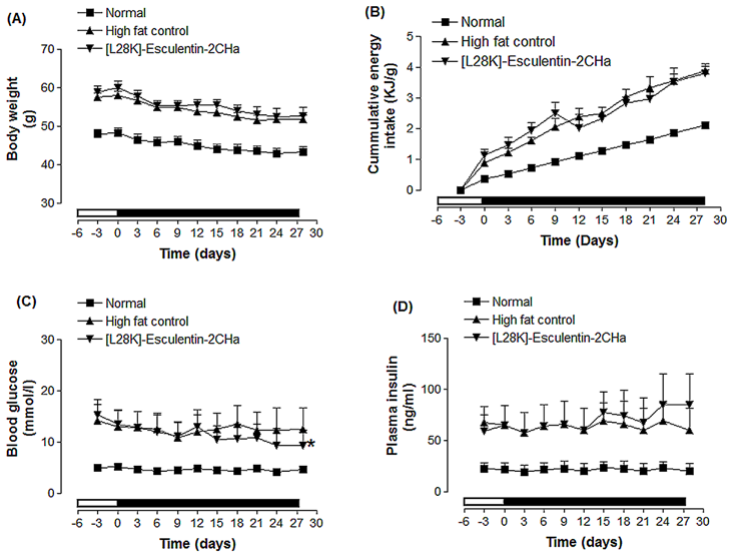
Effects of [L28K]esculentin-2CHa on body weight (A), cumulative energy intake (B), non-fasting plasma glucose (C) and insulin (D) in lean and high-fat fed mice. Parameters were measured 3 days prior to, and every 72 hours during twice-daily treatment (indicated by the black bar) with saline or [L28K]esculentin-2CHa (75 nmol/kg bodyweight) for 28 days. Values are mean ± SEM with n = 8 mice. *P < 0.05, *** P < 0.001 compared to high fat fed control. All parameters were significantly lower in lean mice than high fat fed control mice (P < 0.05 –P < 0.001).

Based on its superior *in vitro* and acute *in vivo* effects, [L28K]esculentin-2CHa was selected for longer-term studies using high fat fed mice. These animals exhibited increased body weight, food intake, plasma glucose and insulin concentrations compared with control animals fed a standard diet (Fig 4). Treatment with [L28K]esculentin-2CHa for 28 days did not affect body weight or food intake but resulted in 25% (P < 0.05, Fig 4C) reduction in non-fasting plasma glucose and a 42% (P < 0.05, Fig 4D) increase in non-fasting plasma insulin compared with saline-treated controls.

### Effects of 28 day administration of [Lys28]esculentin-2CHa on glucose tolerance and insulin-sensitivity

**Fig 5:**
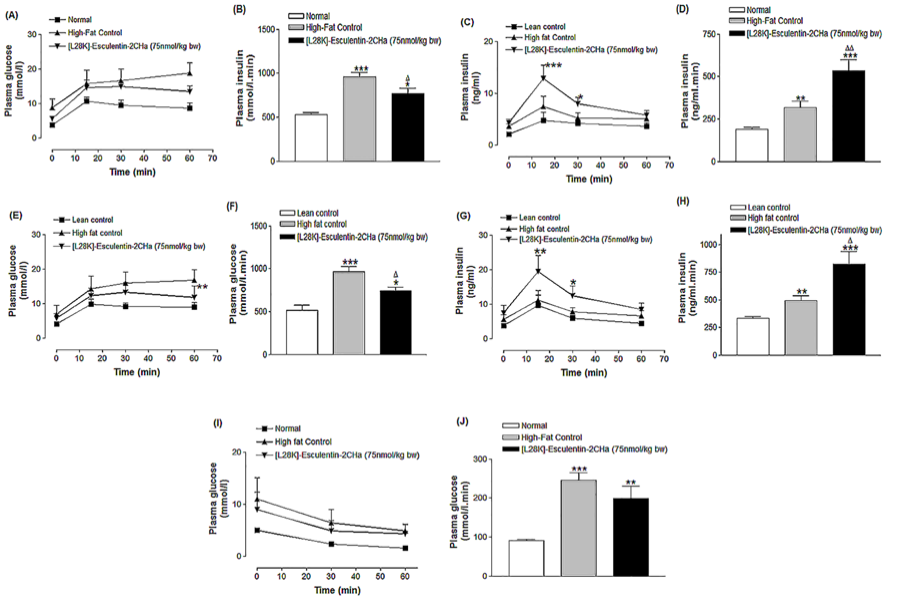
Effects of [L28K]esculentin-2CHa on plasma glucose and insulin concentrations following intraperitoneal (A–D) and oral (E-H) glucose administration (18 mmol/kg body weight) in lean and high-fat fed mice as well as insulin sensitivity (I, J). Insulin sensitivity tests were performed using 25 U/kg body weight of insulin injected intraperitoneally. All tests were conducted following twice-daily treatment of mice with saline or [L28K]esculentin-2CHa (75 nmol/kg body weight) for 28 days. Values are mean ± SEM with n = 8 mice. *P < 0.05, **P < 0.01 and ***P < 0.001 compared with saline-treated lean mice. ^Δ^P < 0.05, ^ΔΔ^P < 0.01 compared with high-fat fed control.

Treatment of high fat fed mice with [L28K]esculentin-2CHa resulted in significant improvement of intraperitoneal glucose tolerance and glucose-stimulated insulin secretion (P < 0.05, Fig 5A–5D). Area under the curve values (AUC 0–60 min) for glucose and insulin concentrations showed an improvement of 20% (P < 0.05, Fig 5B) and 66% (P < 0.01, Fig 5D) respectively. Similarly, the glycaemic excursion following oral glucose (23%, P < 0.01, Fig 5E and 5F) and the accompanying insulin response (68%, P < 0.001, Fig 5G and 5H) were also improved in mice treated with [L28K]esculentin-2CHa. Treatment with the peptide had no significant effect on insulin sensitivity in high fat fed mice (Fig 5I and 5J).

### Effects of 28 day administration of [L28K]esculentin-2CHa on indirect calorimetry, energy expenditure and body composition in high fat fed mice

Oxygen (O_2_) consumption, CO_2_ production and energy expenditure in high fat fed mice increased by 30% (P < 0.001), 22% (P < 0.001) and 27% (P < 0.001) respectively but these parameters together with respiratory exchange ratio were not affected by treatment with [L28K]esculentin-2CHa (S1 Fig). Treatment with [L28K]esculentin-2CHa reversed the loss in bone area but did not affect body fat content and lean body mass in high fat fed mice (S2 Fig). However, saline-treated high fat fed mice exhibited significantly (P < 0.001) reduced bone area (13%, P < 0.001) and increased body fat (2.0-fold, P < 0.001) (S2 Fig) Bone mineral density and bone mineral content were similar in all groups of mice (S2 Fig).

### Effects of 28 day administration of [L28K]esculentin-2CHa on pancreatic weight, insulin content and islets insulin secretory responses of isolated islets

**Fig 6:**
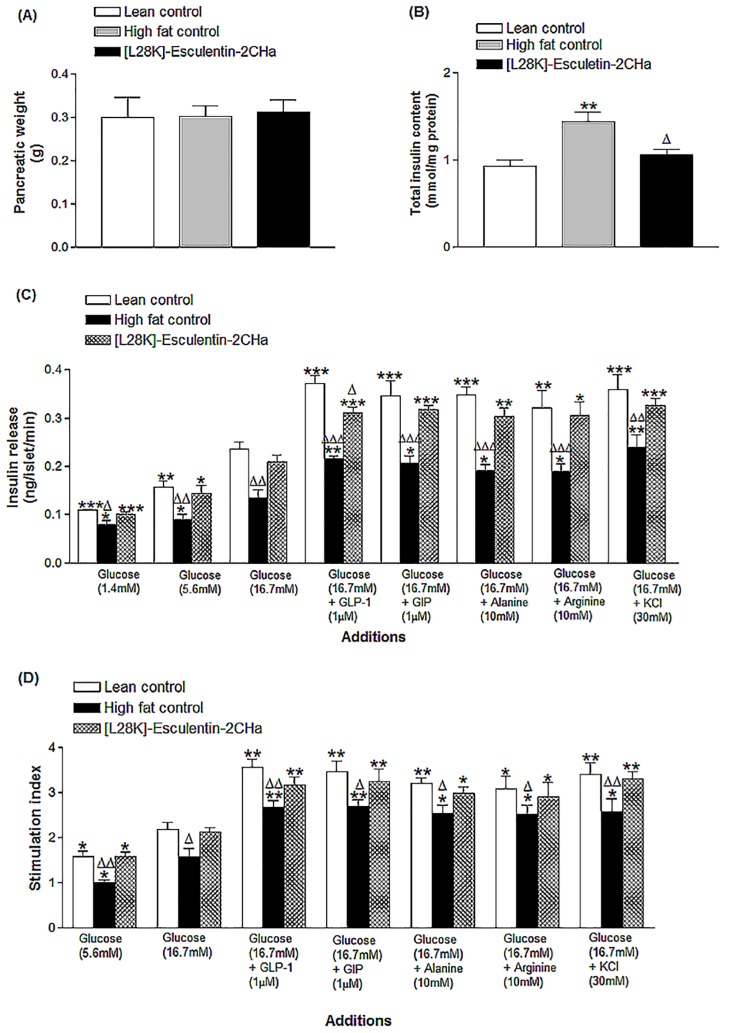
Effects of [L28K]esculentin-2CHa on (A) pancreatic weight, (B) pancreatic insulin content and (C) insulin-secretory responses of islets isolated from lean and high-fat fed mice treated with saline or [L28K]esculentin-2CHa (75 nmol/kg body weight) for 28 days. Values are means ± SEM for 8 mice. For Fig 6B, ***P < 0.001 compared with lean control, ^Δ^P < 0.01 compared with high-fat control. For Fig 6C, *P < 0.05,**P < 0.01, ***P < 0.001 compared with the response of islets isolated from the same group of mice at 16.7 mM glucose; ^Δ^P < 0.05, ^ΔΔ^P < 0.01, ^ΔΔΔ^P < 0.001 compared with the respective response of islets isolated from lean control. For Fig 6D, stimulation index refers to fold-increase in insulin secretion from 1.4 mM glucose to 16.7 mM glucose or from 16.7 mM glucose to that observed in the presence of each secretagogue. *P < 0.05, **P < 0.01 compared with the stimulation index of islets isolated from each group of mice at 16.7mM glucose. ^Δ^P < 0.05, ^ΔΔ^P < 0.01 compared the stimulation index of lean control.

Pancreatic weights were similar in all groups of mice (Fig 6A). However, the greater total pancreatic insulin content of high fat fed mice was reversed by treatment with [L28K]esculentin-2CHa (Fig 6B). Compared with lean control mice, secretory responses of islets isolated from high fat fed mice were significantly decreased to 1.4 (27%, P < 0.05), 5.6 (42%, P < 0.05) or 16.7 mM glucose (43%, P < 0.01), GLP-1 (1 μM, 42%, P < 0.05), GIP (1 μM, 40%, P < 0.05), alanine (10 mM, 45%, P < 0.05), arginine (10 mM, 41%, P < 0.05) and KCl (30 mM, 33%, P < 0.05). However, treatment with [L28K]esculentin-2CHa for 28 days restored the insulin-secretory responses to each of these secretagogues (Fig 6C and 6D).

### Effects of 28 day administration of [L28K]esculentin-2CHa on pancreatic and plasma glucagon content, lipid profile and both renal and liver function

**Fig 7:**
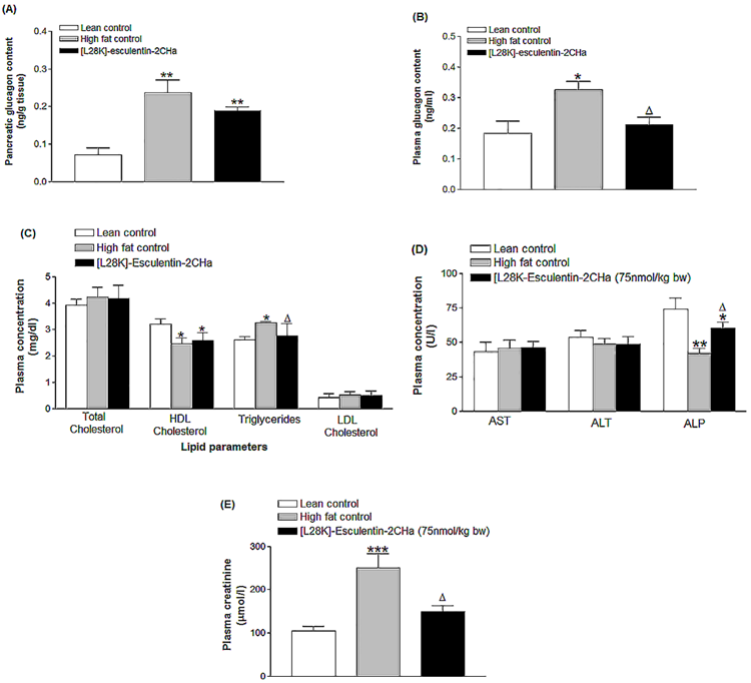
Effects of [L28K]esculentin-2CHa on plasma glucagon content (A), pancreatic glucagon content (B), plasma lipid profile (C), plasma concentrations of AST, ALT and ALP (D) and plasma level of creatinine (E) in lean and high-fat fed mice. Mice were treated with twice-daily injections of saline or [L28K]esculentin-2CHa (75 nmol/kg body weight) for 28 days prior to measurement of biochemical parameters. Values are mean ± SEM with n = 8. *P < 0.05, **P < 0.01, ***P < 0.01 compared with lean control. ^Δ^P < 0.05 compared with high-fat control.

The elevated pancreatic and plasma glucagon levels generated by high fat feeding were reduced by 20% (P < 0.05) and 35% (P < 0.05) in [L28K]esculentin-2CHa-treated mice (Fig 7A and 7B). Plasma total cholesterol were similar in all groups while high fat fed mice exhibited reduced plasma HDL cholesterol (23%, P < 0.05) and elevated plasma triglycerides (1.2-fold, P < 0.05) (Fig 7C). Treatment with [L28K]esculentin-2CHa for 28 days had no effect on HDL cholesterol but significantly (P < 0.05) decreased plasma triglycerides. Plasma levels of AST and ALT were similar in all groups of mice, plasma ALP was decreased (43%, P < 0.01) and creatinine increased (140%, P < 0.001) in high fat fed mice compared with lean controls (Fig 7D and 7E). Administration of [L28K]esculentin-2CHa for 28 days resulted in 43% (P < 0.05, Fig 7D) increase in plasma ALP and 40% (P < 0.05, Fig 7E) decrease in plasma creatinine levels.

### Effects of 28 day administration of [L28K]esculentin-2CHa on islet morphology, beta cell and alpha cell area

**Fig 8:**
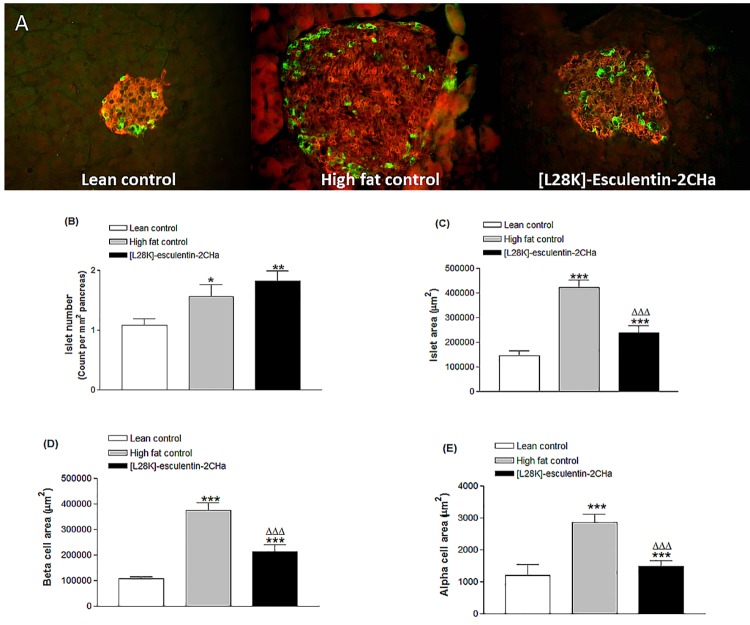
Effects of [L28K]esculentin-2CHa on islet morphology (A) and computed islet number (B), islet area (C), beta cell area (D) and alpha cell area (E) in lean and high-fat fed mice. Mice were treated with twice-daily injections of saline or [L28K]esculentin-2CHa (75 nmol/kg body weight) for 28 days prior to excision of pancreatic tissue for immunohistological analysis. Values are mean ± SEM for 8 observations (~120 islets per group). *P < 0.05, **P < 0.01, ***P < 0.001 compared to lean control. ^ΔΔΔ^P < 0.001 compared to high fat control.

Islets isolated from saline-treated high fat fed mice were significantly larger than those isolated from lean mice (Fig 8A). The number of islets per mm^2^ of pancreas as well as beta and alpha cells area were also significantly (P ≤ 0.05) increased in high fat fed mice (Fig 8B–8E). Treatment with [L28K]esculentin-2CHa did not affect the number of islets per mm^2^ of pancreas but significantly reduced total islet area (44%, P<0.001) as well as beta (43%, P < 0.001) and alpha (48%, P < 0.001) cell areas.

## Discussion

The increasing incidence of type 2 diabetes together with ever mounting challenges posed by associated complications have motivated efforts to discover novel antidiabetic agents with better therapeutic outcomes [23]. The present study provides further evidence for the potentially beneficial actions of the esculentin-2CHa family of peptides and highlights their possible utility in the management of type 2 diabetes. Consistent with observations using several other amphibian host defence peptides [10], esculentin-2CHa stimulates insulin release from BRIN-BD11 cells in a dose-dependent and glucose-responsive manner at concentrations that are non-toxic to the cells. However, magnitude of the effects of the peptide, particularly at the highest concentration tested (3 μM), were appreciably greater than those previously reported for other amphibian host-defence peptides [9]. Indeed, the stimulatory effect of esculetin-2CHa on isolated mouse islets is comparable in magnitude to that of GLP-1 under the same experimental conditions [24,25]. Membrane depolarization and enhanced intracellular Ca^2+^ concentrations together with the reduced insulin release in the presence of diazoxide, verapamil or Ca^2+^ chelation suggest that the insulinotropic action of esculentin-2CHa, in common with other but not all previously identified insulinotropic amphibian skin peptides [5,10], may involve the activation of the K_ATP_-dependent pathway of insulin secretion [26]. This is fully supported by initial patch clam experiments evaluating effects of [Arg4]tigerinin-1R in clonal BRIN-BD11 beta cells (R. C. Moffett et al., unpublished observations). This analogue derived from tigerinin-1R isolated from the skin secretions of *Hoplobatrachus rugulosus* has similar action prolife to esculentin-2CHa. However, effects on other elements of beta cell signal transduction pathways such as glucose metabolism (glycolysis or mitochondrial metabolism), intracellular cyclic AMP generation or activation of phospholipase C should not be ruled out. Indeed, insulin secretion was largely preserved in the presence of diazoxide or verapamil, as well as under Ca^2+^ free conditions, indicating that almost half of the insulin secretory response induced by esculentin-2CHa is dependent neither on K_ATP_ channel closure nor L-type Ca^2+^ channel activation.

We have demonstrated previously in studies using the amphibian peptides pseudin-2 [15], B2RP [27], alyteserin-2a [28] and tigerinin-1R [29] that site-specific amino acid substitutions that result in increasing cationicity may lead to significant enhancement of their *in vitro* insulin releasing actions. In this study, such amino acid substitutions resulted in increased net charge and isoelectric point (Table 1) as well as enhanced insulinotropic potency. However, the responses to a 3μM stimulus of all analogues of esculentin-2CHa tested, except the [L28K] and [C31K] peptides, were lower compared with the native peptide.

Consistent with previous studies [11], mice fed a high fat diet exhibited glucose intolerance and insulin resistance. Acute injection with esculentin-2CHa significantly improved both oral and intraperitoneal glucose tolerance and increased insulin secretion. These *in vivo* actions were greater with the [D28K] and [C31S] analogues while the *in vitro* insulinotropic actions of the other peptide analogues were not replicated *in vivo*. The *in vivo* effects of [L28K] and [C31K]esculentin-2CHa in these animals were more pronounced than those previously reported for other amphibian skin peptides [12–14,30]. Twice-daily administration of [L28K]esculentin-2CHa for 28 days did not affect body weight and food intake but reduced hyperglycaemia and elevated plasma insulin concentrations in the non-fasting state. This was associated with improved glucose tolerance following oral and intraperitoneal glucose administration accompanied by enhanced insulin secretion. These observations are broadly consistent with effects of tigerinin-1R [12], [I10W]-tigerinin-1R [30] and magainin-AM2 [13], suggesting a possible similar spectrum of actions in this animal model of obesity and diabetes. We have previously shown that the antidiabetic effects of magainin-AM2 [13] and [I10W]-tigerinin-1R [30] were accompanied by improvement in insulin sensitivity. However, in the present study, the insulin resistance induced by high fat feeding was not alleviated in mice treated with [L28K]esculentin-2CHa. It is probable, therefore, that the improvement of glucose homeostasis observed in this study is largely due the effects of the peptide on islet cell function. Consistent with this view, the reduced insulin-secretory responses to incretin hormones and other insulin secretagogues observed with islets isolated from saline treated high fat fed mice were reversed by treatment with [L28K]esculentin-2CHa.

Unrestrained hepatic glucose output and elevated glucagon secretion make an important contribution to the development of hyperglycaemia in type 2 diabetes [31]. Interesting, the elevation of pancreatic and plasma glucagon concentrations as well as α-cell area in saline-treated high fat fed mice were normalized by treatment with [L28K]esculentin-2CHa. Although the exact molecular mechanism through which the peptide inhibits α-cell function is not yet known, our results suggest that such effects contribute to the improvement of hyperglycaemia and glucose tolerance. Thus, further studies are required to assess whether [L28K]esculentin-2CHa acts directly on β cells or by indirect effects mediated via enteroendocrine secretions such as GLP-1. Increased islet size accompanying enhanced insulin output and defective β-cell function caused by high fat feeding were also reversed in [L28K]esculentin-2CHa-treated mice. Our preliminary work with analogues of the parent peptide indicate that this may be due to correction of the ratio of beta cell proliferation to apoptosis.

Energy metabolism was not affected by treatment with [L28K]esculentin-2CHa. Similarly, the peptide did not affect adipose tissue deposition, bone mineral content or bone mineral density in high fat fed mice. Bone area and lean body mass were increased in [L28K]esculentin-2CHa-treated mice, but this did not translate to changes in overall body weight. Further studies are required to evaluate long-term impact of [L28K]esculentin-2Cha on circulating cytokines and insulin signalling pathways. Importantly, no adverse effects were observed in mice treated with the peptide, including unchanged circulating concentrations of AST, ALT and ALP together with improved creatinine clearance and lower circulating triglycerides. Although longer term toxicological studies are obviously needed, these observations suggest both benefits and lack of toxicity of [L28K]esculentin-2CHa.

## Conclusions

In conclusion, this study has demonstrated the beneficial effects of esculentin-2CHa family of peptides in mice with diet-induced obesity-diabetes. [L28K]esculentin-2CHa exhibited better in vitro insulinotropic potential compared with the native peptide and significantly improved glucose tolerance and enhanced insulin secretion by positive effects on beta cell function in high fat fed mice. These observations together with the previous demonstrated antimicrobial actions [8] encourage further evaluation of the potential of [L28K]esculentin-2CHa as a novel agent for managing patients with type 2 diabetes and associated microbial infections.

## Supporting Information

S1 Fig Effects of [L28K]esculentin-2CHa on O_2_ consumption (A, B), CO_2_ production (C, D), respiratory exchange ratio (E, F) and energy expenditure (G, H) in lean and high-fat fed treated with saline or [L28K]esculentin-2CHa (75nmol/kg bw) for 28 days. Mice were placed in CLAMS metabolic chambers, and O_2_ consumption or CO_2_ production were measured for 30s at 15min intervals. RER was calculated by dividing VCO_2_ by VO_2_. Energy expenditure was computed using the formula (3.815 + 1.232 x RER) x VO_2_. Values are means ± SEM for 6 mice. **P<0.01, ***P<0.001 compared with saline-treated lean mice. Shaded bar indicates dark phase.
(TIF)

S2 Fig Effects of [L28K]esculentin-2CHa on body composition in normal or high-fat fed mice. Mice were treated with twice-daily injections of saline or [Lys28]esculentin-2CHa (75nmol/kg body weight) for 28 days prior to DEXA scan (A) and computation of data on lean body mass (B), body fat (C, D), bone mineral density (E), bone mineral content (F) and bone area (G). Values are means ± SEM for 8 mice. *P<0.05, **P<0.01, ***P<0.001compared with lean control. ^ΔΔΔ^P<0.01 compared with high fat control.
(TIF)

## References

[1] Nicolas P, El Amri C. The dermaseptin superfamily: a gene-based combinatorial library of antimicrobial peptides. Biochim Biophys Acta. 2009; 1788: 1537–50 10.1016/j.bbamem.2008.09.006 18929530

[2] Conlon JM. Structural diversity and species distribution of host-defense peptides in frog skin secretions. Cell Mol Life Sci. 2011; 68: 2303–15 10.1007/s00018-011-0720-8 21560068 PMC11114843

[3] Bowie JH, Separovic F, Tyler MJ. Host-defense peptides of Australian anurans. Part 2. Structure, activity, mechanism of action, and evolutionary significance. Peptides 2012; 37:174–88 10.1016/j.peptides.2012.06.017 22771617

[4] Conlon JM, Mechkarska M. Host-defense peptides with therapeutic potential from skin secretions of frogs from the family Pipidae. Pharmaceuticals (Basel) 2014; 7: 58–77 24434793 10.3390/ph7010058 PMC3915195

[5] Conlon JM, Mechkarska M, Lukic ML, Flatt PR. Potential therapeutic applications of multifunctional host-defense peptides from frog skin as anti-cancer, anti-viral, immunomodulatory, and anti-diabetic agents. Peptides. 2014; 57: 67–77. 10.1016/j.peptides.2014.04.019 24793775

[6] Pukala TL, Bowie JH, Maselli VM, Musgrave IF, Tyler MJ. Host-defence peptides from the glandular secretions of amphibians: structure and activity. Nat Prod Rep 2006; 23: 368–93 16741585 10.1039/b512118n

[7] Conlon JM, Mechkarska M, Coquet L, Jouenne T, Leprince J, Vaudry H, et al Characterization of antimicrobial peptides in skin secretions from discrete populations of *Lithobates chiricahuensis* (Ranidae) from central and southern Arizona. Peptides 2011; 32:664–9 10.1016/j.peptides.2011.01.018 21262304

[8] Attoub S, Mechkarska M, Sonnevend A, Radosavljevic G, Jovanovic I, Lukic ML, et al Esculentin-2CHa: a host-defense peptide with differential cytotoxicity against bacteria, erythrocytes and tumor cells. Peptides 2013; 39: 95–102 10.1016/j.peptides.2012.11.004 23159562

[9] Heppner KM, Perez-Tilve D. GLP-1 based therapeutics: simultaneously combating T2DM and obesity. Front Neurosci 2015;9:92 10.3389/fnins.2015.00092 25852463 PMC4367528

[10] Ojo OO, Flatt PR, Abdel-Wahab YHA. Insulin-releasing peptides In: Kastin A.J. (Ed.), Handbook of Biologically Active Peptides, Elsevier, San Diego, CA, pp. 364–70

[11] Winzell MS, Ahren B. The high-fat diet-fed mouse: a model for studying mechanisms and treatment of impaired glucose tolerance and type 2 diabetes. Diabetes 2004; 53: S215–S219 15561913 10.2337/diabetes.53.suppl_3.s215

[12] Ojo OO, Srinivasan DK, Owolabi BO, Flatt PR, Abdel-Wahab YHA. Beneficial effects of tigerinin-1R on glucose homeostasis and beta cell function in mice with diet-induced obesity-diabetes. Biochimie 2015; 109:18–26 10.1016/j.biochi.2014.11.018 25483926

[13] Ojo OO, Srinivasan DK, Owolabi BO, Conlon JM, Flatt PR, Abdel-Wahab YHA. Magainin-AM2 improves glucose homeostasis and beta cell function in high-fat fed mice. Biochim Biophys Acta 2015; 1850:80–7 10.1016/j.bbagen.2014.10.011 25459513

[14] Srinivasan DK, Ojo OO, Owolabi BO, Conlon JM, Flatt PR, Abdel-Wahab YHA. The frog skin host-defense peptide CPF-SE1 improves glucose tolerance, insulin sensitivity and islet function and decreases plasma lipids in high-fat fed mice. Eur. J. Pharmacol. 2015; 746:38–47. 10.1016/j.ejphar.2015.06.042 26123844

[15] Abdel-Wahab YHA, Power GJ, Ng MT, Flatt PR, Conlon JM. Insulin-releasing properties of the frog skin peptide pseudin-2 and its [Lys(18)]-substituted analogue. Biol Chem 2008; 389:143–8 10.1515/BC.2008.018 18163889

[16] Mechkarska M, Ojo OO, Meetani MA, Coquet L, Jouenne T, Abdel-Wahab YHA, et al Peptidomic analysis of skin secretions from the bullfrog *Lithobates catesbianus* (Ranidae) identifies multiple peptides with potent insulin-releasing activity. Peptides 2011;32:203–8 10.1016/j.peptides.2010.11.002 21087647

[17] Ojo OO, Abdel-Wahab YHA, Flatt PR, Mechkarska M, Conlon JM. Tigerinin-1R: a potent, non-toxic insulin-releasing peptide isolated from the skin of the Asian frog, *Hoplobatrachus rugulosus* . Diabetes Obes Metab 2011; 13:1114–22 10.1111/j.1463-1326.2011.01470.x 21736689

[18] Lacy PE, Kostianovsky M. Method for the isolation of intact islets of Langerhans from the rat pancreas. Diabetes 1967;16:35–9 5333500 10.2337/diab.16.1.35

[19] Goto M, Maki T, Kiyoizumi T, Satomi S, Monaco AP. An improved method for isolation of mouse pancreatic islets. Transplantation 1985; 40:437–8 2996187 10.1097/00007890-198510000-00018

[20] Flatt PR, Bailey CJ. Abnormal plasma glucose and insulin responses in heterozygous lean (ob/+) mice. Diabetologia 1981; 20:573–7 7026332 10.1007/BF00252768

[21] Albanese CV, Diessel E, Genant HK. Clinical applications of body composition measurements using DXA. J. Clin Densitom 2003; 6, 75–85 12794229 10.1385/jcd:6:2:75

[22] Johnson R, McNutt P, MacMahon S, Robson R. Use of the Friedewald formula to estimate LDL-cholesterol in patients with chronic renal failure on dialysis. Clin Chem 1997, 43, 2183–2184 9365406

[23] Blonde L. Current challenges in diabetes management. Clin Cornerstone 2005;7:S6–1 16545737 10.1016/s1098-3597(05)80084-5

[24] Gault VA, Porter DW, Irwin N, et al Comparison of sub-chronic metabolic effects of stable forms of naturally occurring GIP(1–30) and GIP(1–42) in high-fat fed mice. J Endocrinol 2011; 208:265–71 10.1530/JOE-10-0419 21212092

[25] Fridolf T, Ahrén B. GLP-1(7–36) amide stimulates insulin secretion in rat islets: studies on the mode of action. Diabetes Res. 1991; 16:185–91 1802486

[26] Koster JC, Permutt MA, Nichols CG. Diabetes and insulin secretion: the ATP-sensitive K+ channel (K ATP) connection. Diabetes 2005; 54:3065–72 16249427 10.2337/diabetes.54.11.3065

[27] Abdel-Wahab YH, Patterson S, Flatt PR, Conlon JM. Brevinin-2-related peptide and its [D4K] analogue stimulate insulin release in vitro and improve glucose tolerance in mice fed a high fat diet. Horm Metab Res 2010; 42: 652–6 10.1055/s-0030-1254126 20496306

[28] Ojo OO, Abdel-Wahab YHA, Flatt PR, Conlon JM. Insulinotropic actions of the frog skin host-defense peptide alyteserin-2a: a structure-activity study. Chem Biol Drug Des 2013; 82: 196–204 10.1111/cbdd.12151 23742240

[29] Srinivasan D, Ojo OO, Abdel-Wahab YHA, Flatt PR, Guilhaudis L, Conlon JM. Insulin-releasing and cytotoxic properties of the frog skin peptide, tigerinin-1R: a structure-activity study. Peptides 2014; 55:23–31 10.1016/j.peptides.2014.02.002 24530698

[30] Srinivasan DK, Ojo OO, Owolabi BO, Conlon JM, Flatt PR, Abdel-Wahab YHA. [I10W]tigerinin-1R enhances both insulin sensitivity and pancreatic beta cell function and decreases adiposity and plasma triglycerides in high-fat mice. Acta Diabetol (In Press) 26138324 10.1007/s00592-015-0783-3

[31] D’Alessio D. The role of dysregulated glucagon secretion in type 2 diabetes. Diabetes Obes Metab 2011; 13:126–32 10.1111/j.1463-1326.2011.01449.x 21824266

