# Supplementary figures and images for: Esculentin-2CHa-Related Peptides Modulate Islet Cell Function and Improve Glucose Tolerance in Mice with Diet-Induced Obesity and Insulin Resistance
Source: PLoS One. 2015 Oct 29;10(10):e0141549. doi: 10.1371/journal.pone.0141549 (PMC4626215; doi:10.1371/journal.pone.0141549)

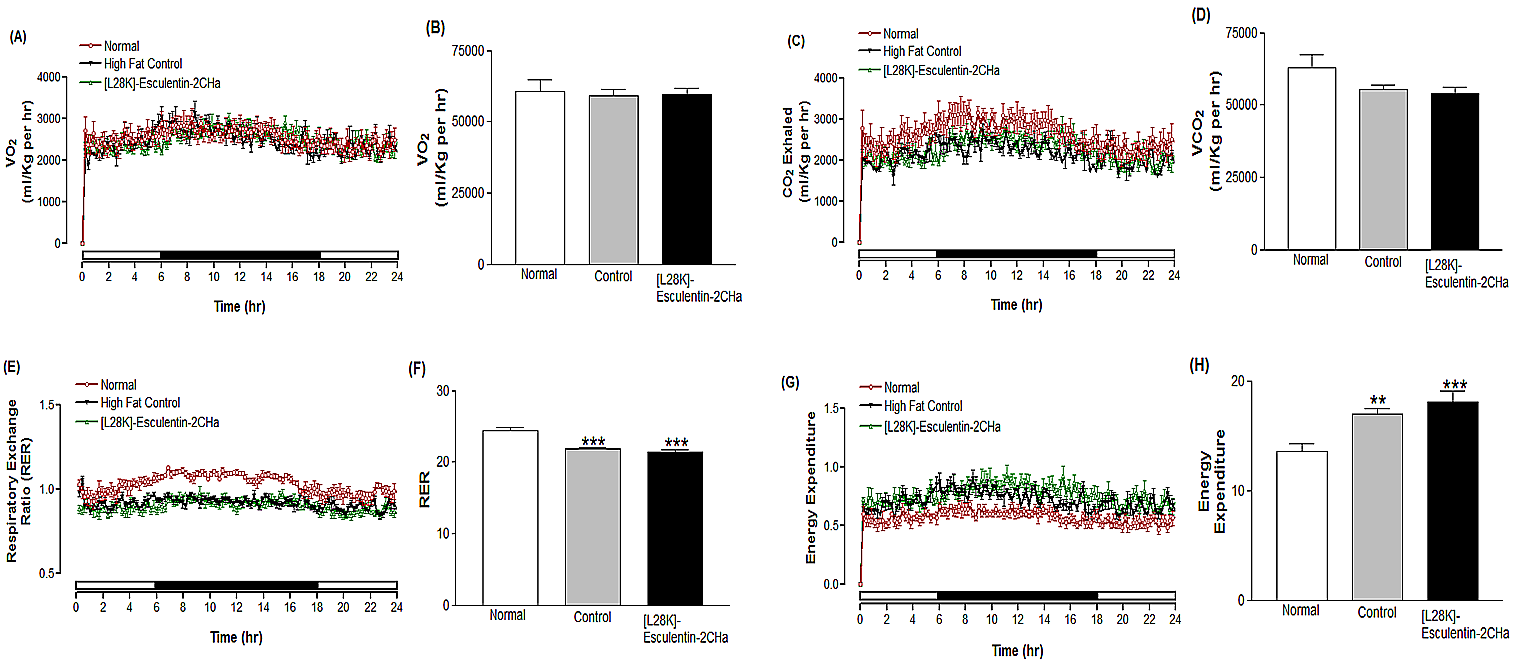

Supplement: S1 Fig — Mice were placed in CLAMS metabolic chambers, and O2 consumption or CO2 production were measured for 30s at 15min intervals. RER was calculated by dividing VCO2 by VO2. Energy expenditure was computed using the formula (3.815 + 1.232 x RER) x VO2. Values are means ± SEM for 6 mice. **P<0.01, ***P<0.001 compared with saline-treated lean mice. Shaded bar indicates dark phase. (TIF) [file pone.0141549.s001.tif]

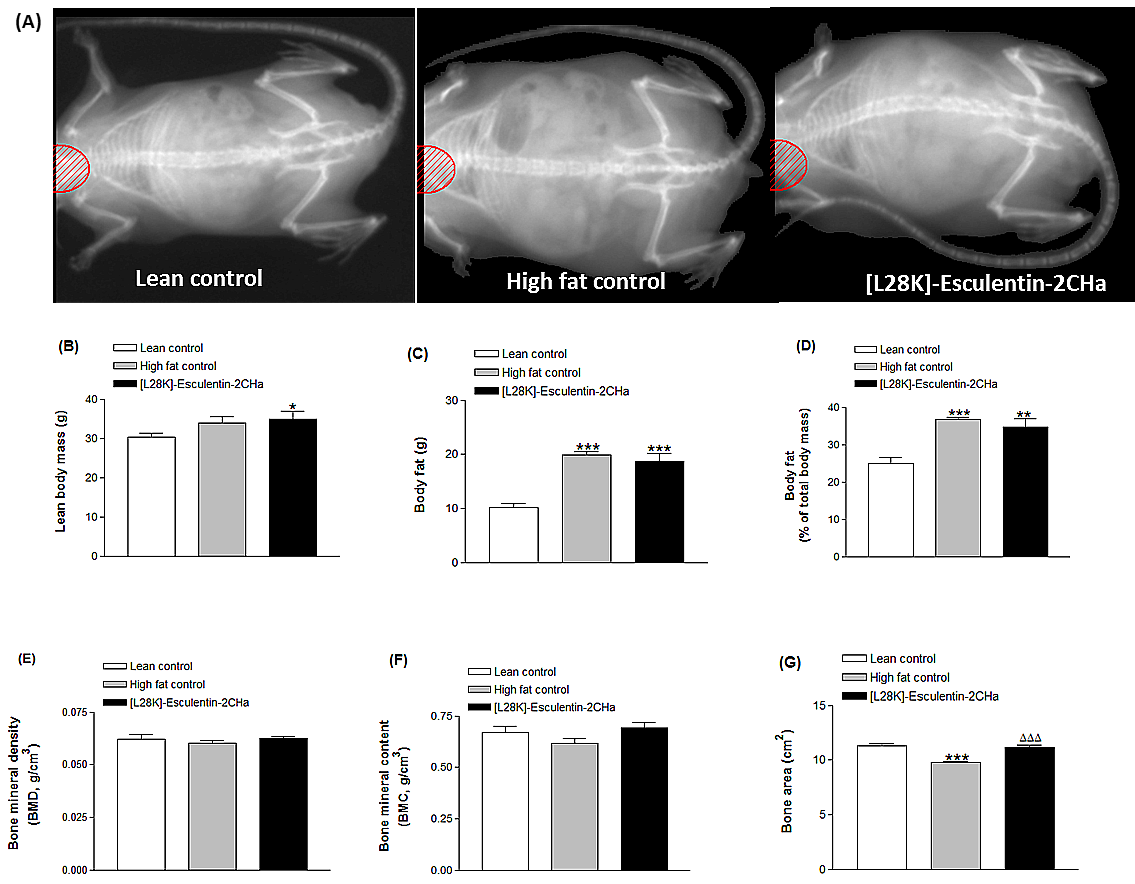

Supplement: S2 Fig — Mice were treated with twice-daily injections of saline or [Lys28]esculentin-2CHa (75nmol/kg body weight) for 28 days prior to DEXA scan (A) and computation of data on lean body mass (B), body fat (C, D), bone mineral density (E), bone mineral content (F) and bone area (G). Values are means ± SEM for 8 mice. *P<0.05, **P<0.01, ***P<0.001compared with lean control. ΔΔΔP<0.01 compared with high fat control. (TIF) [file pone.0141549.s002.tif]
